# Molecular mechanisms of long noncoding RNAs on gastric cancer

**DOI:** 10.18632/oncotarget.6926

**Published:** 2016-01-17

**Authors:** Tianwen Li, Xiaoyan Mo, Liyun Fu, Bingxiu Xiao, Junming Guo

**Affiliations:** ^1^ Department of Biochemistry and Molecular Biology, and Zhejiang Key Laboratory of Pathophysiology, Ningbo University School of Medicine, Ningbo, China; ^2^ Department of Hepatology, Ningbo No.2 Hospital, Ningbo, China

**Keywords:** lncRNA, gastric cancer, miRNA, ceRNA, gene expression

## Abstract

Long noncoding RNAs (lncRNAs) are non-protein coding transcripts longer than 200 nucleotides. Aberrant expression of lncRNAs has been found associated with gastric cancer, one of the most malignant tumors. By complementary base pairing with mRNAs or forming complexes with RNA binding proteins (RBPs), some lncRNAs including GHET1, MALAT1, and TINCR may mediate mRNA stability and splicing. Other lncRNAs, such as BC032469, GAPLINC, and HOTAIR, participate in the competing endogenous RNA (ceRNA) network. Under certain circumstances, ANRIL, GACAT3, H19, MEG3, and TUSC7 exhibit their biological roles by associating with microRNAs (miRNAs). By recruiting histone-modifying complexes, ANRIL, FENDRR, H19, HOTAIR, MALAT1, and PVT1 may inhibit the transcription of target genes *in cis* or *trans*. Through these mechanisms, lncRNAs form RNA-dsDNA triplex. CCAT1, GAPLINC, GAS5, H19, MEG3, and TUSC7 play oncogenic or tumor suppressor roles by correlated with tumor suppressor P53 or onco-protein c-Myc, respectively. In conclusion, interaction with DNA, RNA and proteins is involved in lncRNAs’ participation in gastric tumorigenesis and development.

## INTRODUCTION

Generally defined as transcripts longer than 200 nucleotides, long noncoding RNAs (lncRNAs) are lack of significant open reading frames [1]. According to their location and orientation, lncRNAs are classified as intergenic lncRNAs (lincRNAs), genic and intragenic lncRNAs [2]. In nucleus, lncRNAs mainly modulate gene transcription and mRNA splicing; while they impact RNA stability and microRNA (miRNA) activity in cytoplasm [3].

Among the malignant tumors, gastric cancer remains the fourth most prevalent one and the second leading mortality [4]. Over the last decade, remarkable progresses about gastric cancer-associated lncRNAs have been achieved. LncRNAs are involved in several tumor signaling pathways such as Notch, mTOR, NF-κb, and Wnt [5, 6]. They manipulate cell proliferation, migration, apoptosis, invasion, tumorigenicity, cell cycle, and metastasis (Table 1). Besides, accumulated evidences suggest that the aberrant expressions of lncRNAs have clinical significances in gastric cancer diagnosis [7-17]. They are associated with clinicopathological factors including metastasis, invasion, TNM stage, prognosis, tumor size, and differentiation of patients with gastric cancer (Table 2). Among them, the most proportion are involved in metastasis and invasion (61.70% and 53.19%, respectively). These gastric cancer-associated lncRNAs may be used as biomarkers for indicating metastasis of gastric cancer [18-24].

**Table 1:** Roles of lncRNAs in gastric cancer cells

Function	LncRNAs	Number	Percentage (%)
Proliferation	ANRIL, BC032469, CCAT1, FENDRR, GAPLINC, GAS5, GHET1, H19, HOTAIR, LEIGC, Linc00152, LSINCT5, MALAT1, MEG3, nc886, PVT1, SPRY4-IT1, TINCR, TUSC7, UBC1, UCA1	21	72.41
Migration	BC032469, CCAT1, FENDRR, FRLnc1, H19, HOTAIR, HULC, LEIGC, Linc00152, MALAT2, SDMGC, SPRY4-IT1, UBC1	13	44.83
Apoptosis	ANRIL, CCAT1, GAS5, H19, HOTAIR, HULC, Linc00152, MEG3, nc886, PVT1, TINCR	11	37.93
Invasion	ATB, FENDRR, GAPLINC, H19, HOTAIR, HULC, Linc00152, SDMGC, SPRY4-IT1, UBC1	10	34.48
Tumorigenicity	ANRIL, BC032469, GAS5, GHET1, H19, HOTAIR, LEIGC, PVT1, TINCR	9	31.03
Cell cycle	AC130710, CCAT1, GAS5, Linc00152, MALAT1, MEG3, PVT1, TINCR	8	27.59
Metastasis	FENDRR, FRLnc1, H19	3	10.34
Total		29	100

**Table 2:** LncRNAs and their clinical association of patients with gastric cancer

Clinicopathological factor	LncRNAs	Number	Percentage (%)
Metastasis	AA174084, AC096655.1-002, AC130710, BANCR, CCAT1, CCAT2, FENDRR, FER1L4, FRLnc1, GACAT2, GACAT3, GAPLINC, GAS5, H19, HIF1A-AS2, HOTAIR, HULC, LET, LSINCT5, MALAT1, MALAT2, ncRuAR, RP11-789C1.1, SPRY4-IT1, SUMO1P3, TINCR, UBC1, XLOC_010235, ZMAT1 transcript variant 2	29	61.70
Invasion	AA174084, AI364715, ATB, BANCR, CCAT1, FENDRR, FER1L4, GACAT2, GAS5, GHET1, H19, HIF1A-AS2, HOTAIR, LET, Linc00152, LSINCT5, MEG3, ncRuAR, PVT1, SPRY4-IT1, SUMO1P3, TINCR, TUSC7, UCA1, ZMAT1 transcript variant 2	25	53.19
TNM stage	AC096655.1-002, AC130710, BANCR, BM7402401, CCAT1, FENDRR, FER1L4, GACAT3, GAS5, H19, HIF1A-AS2, HOTAIR, HULC, LET, LSINCT5, MEG3, PVT1, RP11-789C1.1, SPRY4-IT1, TINCR, UBC1, UCA1, XLOC_010235, ZMAT1 transcript variant 2	24	51.06
Prognosis	AC138128.1, ATB, BANCR, BM7402401, CCAT2, DRAIC, FENDRR, GAS5, GHET1, H19, HIF1A-AS2, HOTAIR, LET, LSINCT5, MALAT2, MEG3, PVT1, RP11-789C1.1, SPRY4-IT1, TINCR, UBC1, XLOC_010235, ZMAT1 transcript variant 2	23	48.94
Tumor size	AC130710, AI364715, CCAT1, DKFZP434K028, FER1L4, GACAT3, GAPLINC, GAS5, GHET1, HOTAIR, Linc00152, LSINCT5, MEG3, ncRuAR, RPL34-AS1, SPRY4-IT1, SUMO1P3, UBC1, UCA1	19	40.43
Differentiation	ABHD11-AS1, AC096655.1-002, AI364715, HOTAIR, SUMO1P3, TUSC7, UCA1	7	14.89
Total		47	100

In this article, we summarized the molecular mechanisms of lncRNAs on gastric cancer. By interacting with DNA, RNA, and proteins, lncRNAs play crucial roles in gastric tumorigenesis and development.

## INTERACTION WITH DNA

LncRNAs may combine with histone-modifying complexes and then target on DNA [25]. For example, combining with histone-modifying complexes, forkhead box F1 (*FOXF1*) adjacent non-coding developmental regulatory RNA (FENDRR) anchors to targeted promoter fragments [26]. HOX transcript antisense RNA (HOTAIR) also occupies targeted double-strained DNA (dsDNA) [27]. Additionally, the lncRNA transcribed from the minor promoter of the human dihydrofolate reductase (*DHFR*) gene combines with the key regulatory region of its host gene in a triple helical model [28]. These triplex structures can be served as a specific recognition mechanism between lncRNA and genomic DNA. Theoretically, these are involved in specificities and affinities. Triplexes created by lncRNA and genomic DNA may decisively result in targeting specificity. Favorable chromatin conformation may contribute to the affinity [29].

## INTERACTION WITH RNA

### Interaction with mRNA

**Figure 1:**
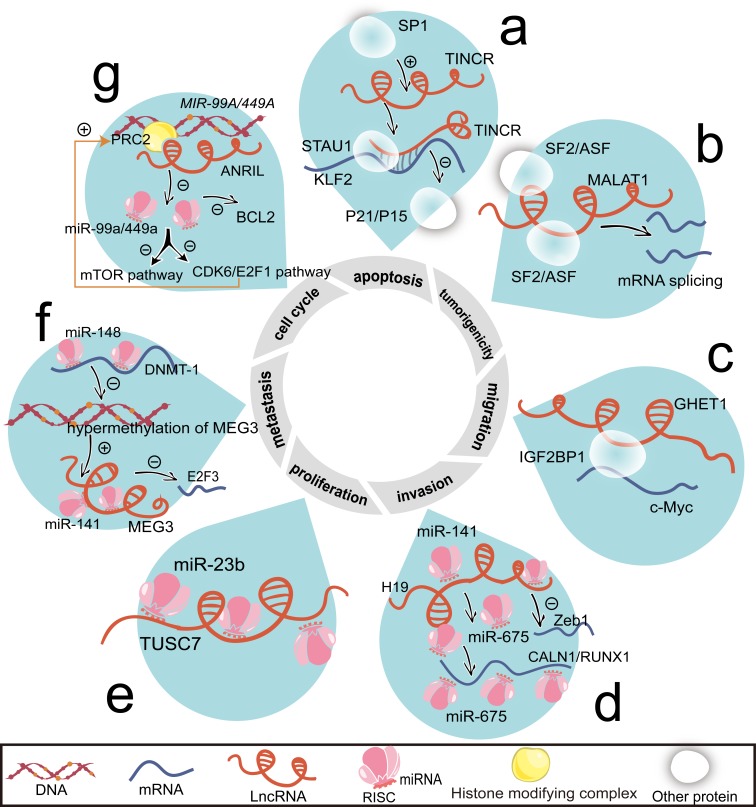
Associated with mRNAs and miRNAs, lncRNAs regulate cell proliferation, cell cycle, apoptosis, invasion, migration, metastasis, and tumorigenicity **a.** Induced by SP1, TINCR duplexes with KLF2 mRNA. STAU1 recognizes the double strained RNA and accelerates KLF2 mRNA degradation. Then, KLF2 transcriptionally activates *P21/P15* and elevates protein levels of P21/P15. **b.** MALAT1 changes the distribution of SF2/ASF and then indirectly influences mRNA splicing. **c.** By binding to IGF2BP1, GHET1 promotes c-Myc mRNA stability. **d.** By binding with miR-141, H19 inhibits ZEB1 mRNA level. H19 also generates to miR-675, which suppresses the expression of CALN1 and RUNX1. **e.** TUSC7 is a negative target of miR-23b. **f.** miR-148a negatively regulates DNMT-1 expression, thus reducing the hypermethylation of MEG3 promoter region. Loss of hypermethylation enhances the transcription of MEG3. Through direct interacting with miR-141, MEG3 downregulates E2F3 mRNA expression. **g.** By epigenetically silencing *MIR-49A/MIR-449A*, ANRIL promotes mTOR and CDK6/E2F1 pathway that feedback enhances ANRIL expression. And miR-449a inhibits BCL2 expression. Abbreviations: CALN1, calneuron 1; E2F3, E2F transcription factor 3; mTOR, mechanistic target of rapamycin; P15, cyclin-dependent kinase inhibitor 2B; RUNX1, runt domain transcription factor 1; SP1, nuclear transcription factor.

The base paring is formed between protein-coding transcript and the complementary lncRNA, the natural antisense transcript (NAT). Interestingly, the RNA duplex may be created by incompletely base pairing between Alu elements in a targeted mRNA and the complementary sequence harbored in an lncRNA. Staufen 1 (STAU1) protein recognizes the dsRNA binding sites and then results in mRNA degradation. This type of lncRNAs is called 1/2-STAU1-binding site RNAs [30]. And the process is termed as staufen-mediated mRNA decay (SMD) [31]. For instance, tissue differentiation-inducing non-protein coding RNA (TINCR) impairs the stability and expression of Krüppel-like factor 2 (KLF2) mRNA through SMD (Figure 1a). TINCR enables the stabilization of mRNAs by duplexing with mRNAs containing TINCR box motif [32].

Irrespective of the direct interaction, the indirect mode between lncRNAs and their targeted mRNAs is illustrated by metastasis-associated lung adenocarcinoma transcript 1 (MALAT1) (Figure 1b) and gastric carcinoma proliferation enhancing transcript 1 (GHET1) (Figure 1c). By binding with associated proteins, MALAT1 and GHET1 alter splicing or stability of mRNAs [33-35]. The function of serine/arginine splicing factors (SF2/ASF) relies on MALAT1 (Figure 1b). Formed by a bipartite triple helix, MALAT1 promotes malignancy [36]. The fragment nearly 3′ end of MALAT1 may be in charge of the metastatic potential [37].

GHET1 potentiates the combination between insulin-like growth factor 2 mRNA binding protein 1 (IGF2BP1) and c-Myc mRNA (Figure 1c). Albeit the short recognition element (CAUH, H = A, U, or C) of IGF2BP1 is extensively mapping to 8400 coding genes genome-wide [38]. Remarkably, the motif CAUH is too short to format the secondary structure.

About a quarter of lncRNAs are consist of one or more Alu elements [30]. Inverted Alu repeats form long stable stem-loop structures and activate the post-transcription and translation [39, 40]. Interestingly, the long inverted repeats flanking the mouse *Sry* gene enable the formation of a circular RNA [41]. Apart from the secondary structure in repeat elements, it is likely that the special architectures of lncRNAs accelerate their functions. Therefore, high order structures of lncRNAs may conduce to the specific recognization with proteins and nucleic acids.

### Interaction with miRNAs

By assembly of RNA induced silencing complex (RISC), miRNAs lead mRNA degradation [42]. Studies showed that some lncRNAs are the precursors of miRNAs [43, 44]. These mean that lncRNAs may affect mRNA degradation in a indirect way.

miR-675, processed from its precursor (imprinted maternally expressed transcript, H19), has the properties of promoting proliferation and epithelial-mesenchymal transition (EMT) (Figure 1d). H19 is capable of enhancing gastric carcinogenesis [45]. Plasma H19 levels in patients with gastric cancer are significantly higher than those in healthy controls [46].

Besides, lncRNAs’ expressions may be repressed by miRNAs [47-49]. The situations in gastric cancer are sketched by tumor suppressor candidate 7 (TUSC7)/miR-23b (Figure 1e), H19/miR-141 (Figure 1d), and maternally expressed 3 (MEG3)/miR-141 (Figure 1f).

Apart from miR-141, miR-148a shows an inhibitory effect on the expression of DNA methyltransferase 1 (DNMT-1) and thus induces the overexpression of MEG3 [50]. By recruiting polycomb repressive complex 2 (PRC2) to the functional sites [51], lncRNA-CDKN2B antisense RNA 1 (ANRIL) silences *MIR-99A and MIR-449A* (Figure 1g).

The examples about miRNAs’ regulating lncRNAs via epigenetic modification, *vice versa*, can also be found in other parts of this article.

### LncRNAs acting as ceRNAs

Emerging evidences suggest that lncRNAs may participate in competitive endogenous RNA (ceRNA) network [52], in which lncRNAs cross talk with other RNAs by sharing miRNAs.

**Figure 2:**
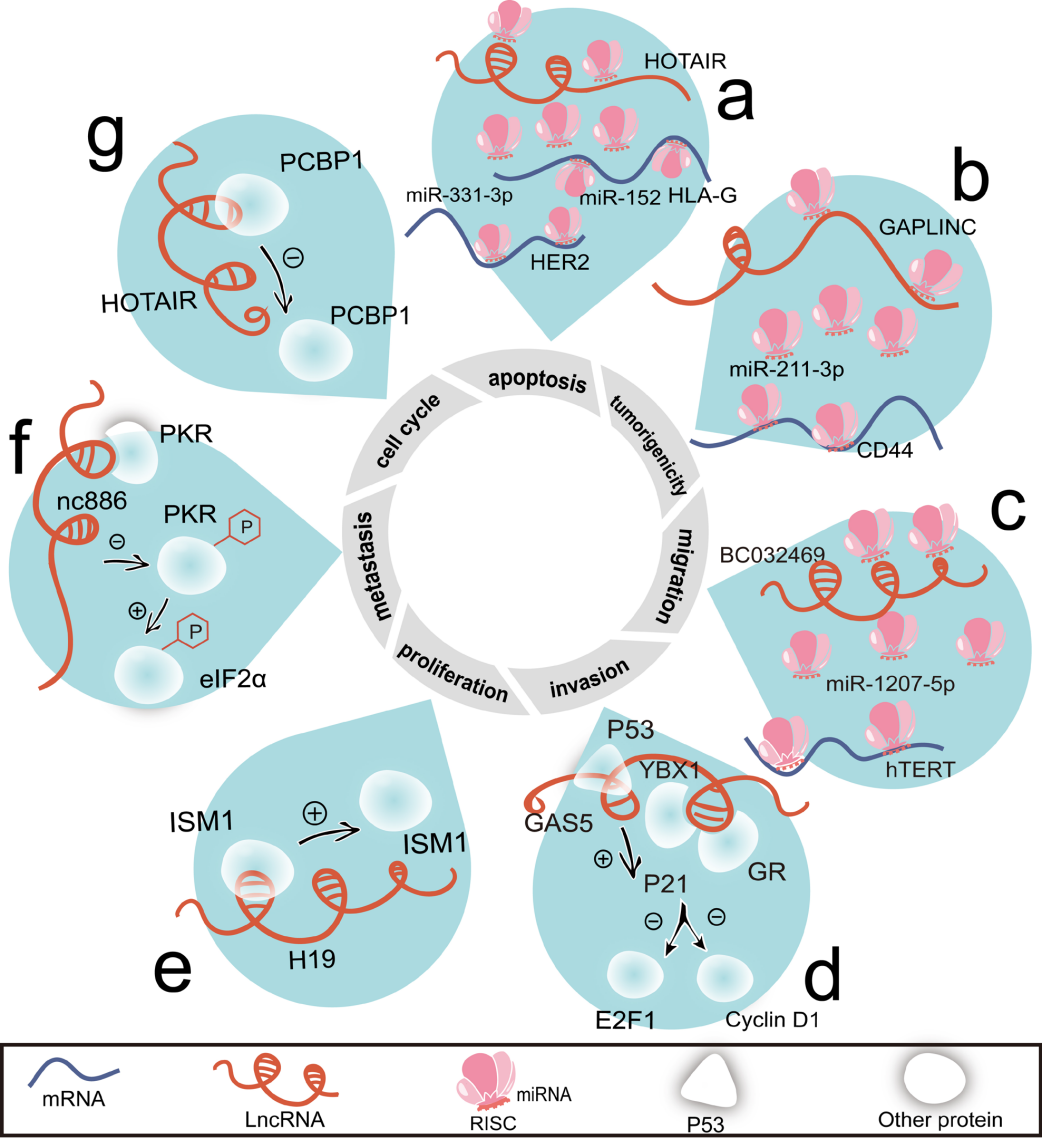
LncRNAs regulate cell proliferation, cell cycle, apoptosis, invasion, migration, metastasis, and tumorigenicity by interacting with proteins or acting as ceRNAs **a.** There are two ceRNA networks associated with HOTAIR, HOTAIR/miR-331-3p/HER2 mRNA and HOTAIR/miR-152/HLA-G mRNA. **b.** By sharing miR-211-3p, GAPLINC and CD44 become a pair of ceRNAs. **c.** Through sponging miR-1207-5p, BC032469 competes with hTERT mRNA. **d.** By binding with GR, YBX1 and P53, GAS5 elevates P21 level and represses the levels of E2F1 and cyclin D1. **e.** Combing with ISM1, H19 positively regulates its protein level. **f.** Nc886 represses the phosphorylation of PKR, thus blocking the phosphorylation of eIF2α. **g.** Binding with PCBP1, HOTAIR attenuates its protein level. Abbreviations: eIF2α, eukaryotic translation initiation factor 2, subunit 1 α.

Plasma HOTAIR levels in gastric cancer patients are higher than health controls [46]. Through the competitive ‘sequestration’ of miR-331-3p/miR-124, HOTAIR and erythroblastic leukemia viral oncogene homolog 2 (HER2) mRNA become a pair of ceRNAs in gastric cancer (Figure 2a). HER2 triggers malignant phenotype in gastric cancer upon drug resistant and EMT [53, 54]. HOTAIR may be a novel target for HER2 positive patients who herald the high metastatic potential and poor survival [55]. Similarly, CD44 is a well-characterized glycoprotein involved in cancer metastasis [56]. Gastric adenocarcinoma predictive lincRNA (GAPLINC)/miR-211-3p/CD44 axis may also be applied to against metastasis (Figure 2b). Human leukocyte antigen-G (HLA-G) and human telomerase reverse transcriptase (hTERT) are both antigens expressed upon tumor cells [57, 58]. The positive correlations of HOTAIR/HLA-G (Figure 2a) and lncRNA-BC032469/hTERT (Figure 2c) provide potential immunotherapy targets on gastric cancer [59, 60].

Based on miRNA and lncRNA microarray data of gastric cancer, our group constructed a ceRNA network interlaced by gastric cancer-associated lncRNAs, miRNAs, and mRNAs [61]. For instance, fer-1-like family member 4, pseudogene (FER1L4), gastric cancer associated transcript 1 (GACAT1), and H19 may upregulate or downregulate the expression of PTEN, RB1, RUNX1, VEGFA, CDKN1A, E2F1, HIPK3, IL-10, or PAK7 by sharing miR-106a-5p [61, 62]. This deduction suggests that blocking the associations between lncRNAs and their partners (RNAs or proteins) may enable to cascade a significant effect.

## INTERACTION WITH PROTEINS

### Interaction with histone-modifying complexes

**Figure 3:**
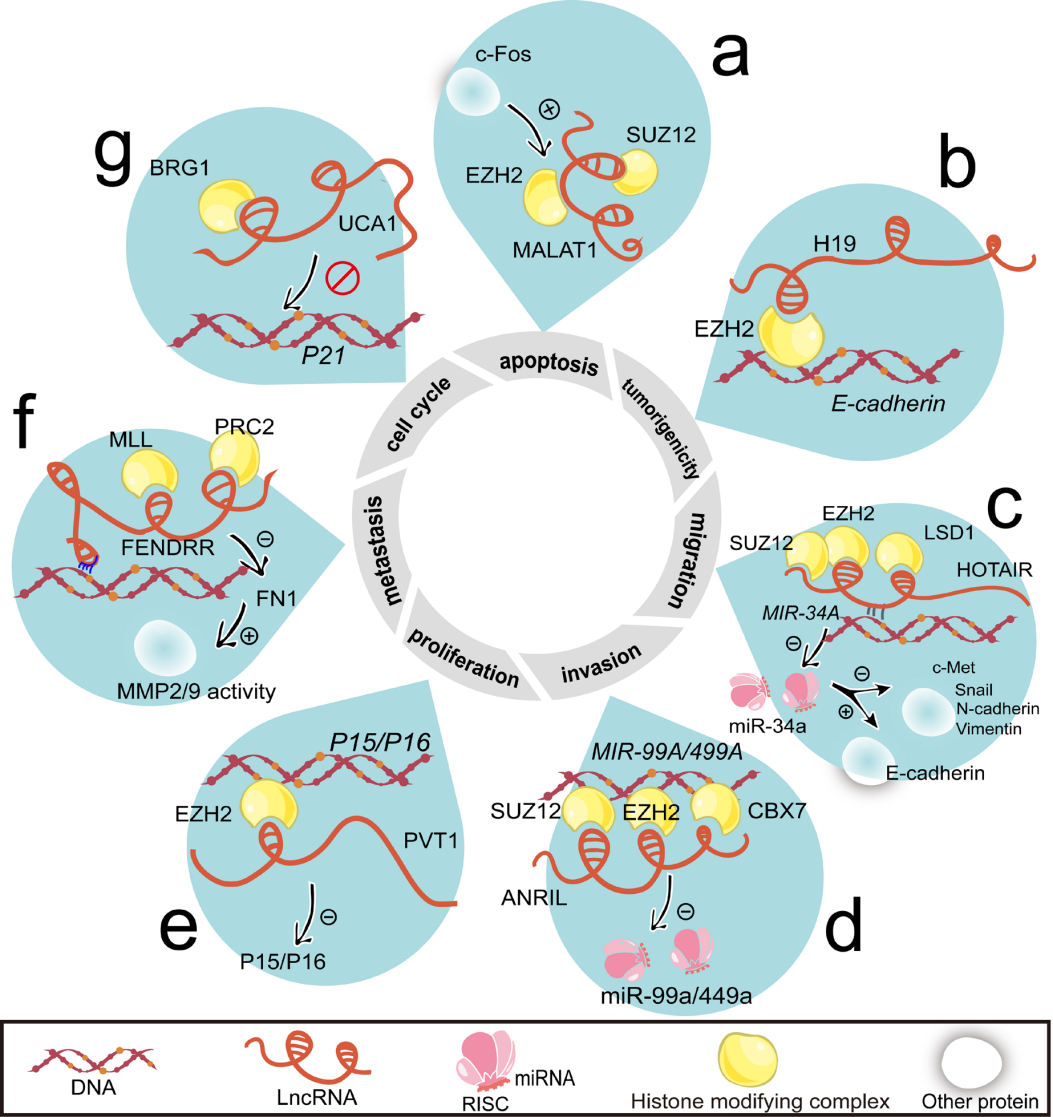
LncRNAs regulate cell proliferation, cell cycle, apoptosis, invasion, migration, metastasis, and tumorigenicity by histone modification **a.** Activated by c-Fos, MALAT1 binds with EZH2 and SUZ12. **b.** H19 guides EZH2 to the *E-cadherin* promoter loci. **c.** Through the formation of dsDNA/RNA triplex, HOTAIR recruits SUZ12 and EZH2 to the *MiR-34A* loci and then silences the transcription of miR-34a. HOTAIR also forms complex with LSD1. HOTAIR attenuates c-Met, Snail, N-cadherin and Vimentin protein level while upregulates E-cadherin protein level. All of them are targets of miR-34a. **d.** ANRIL suppresses miR-49a/449a expression by the recruitment of EZH2 and SUZ12. ANRIL also combines with CBX7. **e.** The *P15/P16* is silenced by the occupancy of EZH2, which is recruited by PVT1. **f.** Using its complementary fragment, FENDRR anchors with target DNA and guides MLL and PRC2 to the targeted genes. Additionally, FENDRR decreases MMP2/MMP9 activity by reducing FN1. **g**. Combining with BRG1, UCA1 impairs BRG1's ability to bind with *P21* promoter. Abbreviations: FN1, fironectin 1; MMP2/9, matrix metalloproteinase 2/9.

As many as 38% lincRNAs cooperate with at least one of multiple histone-modifying complexes [63]. This suggests that one lncRNA may harbor several types of binding elements for chromatin modifiers. The specific locus is subject to the ensuing histone modification caused by the occupancy of histone-modifying complexes [64]. Apart from HOTAIR [65], PRC2 is found to communicate with other lncRNAs including ANRIL [51, 66], FENDRR [67], plasma-cytoma variant translocation 1 (PVT1) [68], MALAT1 (Figure 3a) and H19 (Figure 3b).

HOTAIR is the first demonstrated lncRNA coordinating gene silencing via assembly of PRC2 [69]. The structural domains of HOTAIR formed in its 5′ region and 3′ region are bound to enhancer of zeste 2 (EZH2, PRC2 subunit) and lysine specific demethylase 1 (LSD1), respectively [70]. HOTAIR preferentially occupies a GA-rich DNA motif to enable the formation of RNA:dsDNA triplex. This occurs independently of EZH2 [27]. Simultaneously, HOTAIR is required for the occupancy of suppressor of zeste 12 homolog (SUZ12, PRC2 subunit) on *MIR-34A* loci (Figure 3c). With the metastasis potential, H19 and HOTAIR epigenetically modify their targeted genes including *E-cadherin* (Figure 3b, 3c). Remarkably, the 1062 nt region at the 5′ end of H19 is indispensable [71].

The manner of ANRIL to silence *P15/INK4a* and *P16/INK4b in cis* (Figure 3d) is different to that of PVT1 to *P15/INK4a* and *P16/INK4b in trans* (Figure 3e). ANRIL and PVT1 behave in the same pattern by EZH2 occupancy in the same site. The upregulated EZH2 enforces gastric cancer cell proliferation [68]. As an antisense lncRNA emanating from *INK4b/ARF/INK4a*, ANRIL is not only a cis-acting lncRNA, but also recruits PRC2 to the distant loci of *MIR-99A/MIR-449A* in gastric cancer (Figure 3d). Furthermore, by downregulating serine/threonine kinase mTOR and cyclin-dependent kinase 6 (CDK6)/ E2F transcription factor 1 (E2F1) pathways, miR-99a/miR-449a indirectly induce the expression of ANRIL [55]. This is a positive feedback loop. Interestingly, CDK6 represents as a *bona fide INK4b/ARF/INK4a* downstream effector. Meanwhile, ANRIL tethers PRC1 subunit chromobox homolog 7 (CBX7) to target genes (Figure 3d).

LncRNAs also recruit activating chromatin modifiers such as lysine (K)-specific methyltransferase 2A (MLL) [26]. FENDRR acts as a propeller for metastasis in gastric cancer [22]. The FENDRR/PRC2 complex may antagonize FENDRR/MLL (Figure 3f). However, rare change caused by FENDRR in its host gene *FOXF1* is observed in gastric cancer [22]. Regardless of promotion effect of lncRNAs on the binding of chromatin-remolding complex, lncRNA-urothelial cancer associated 1 (UCA1) impairs SMARCA4/BRG1 binding to cyclin-dependent kinase inhibitor 1A (*P21*) promoter (Figure 3g). The full name of SMARCA4 is SWI/SNF related, matrix associated, actin-dependent regulator of chromatin, subfamily A, member 4 [78]. It can be noted that BRG1 mediates eviction of the PRC1 and PRC2 at *INK4b/ARF/INK4a* locus [72]. PRC2 is recruited by ANRIL and PVT1 to the same site.

### Interaction with P53 and c-Myc

Among the transcription factors (TFs) activating carcinogenesis, P53 and c-Myc represent potent inducers. The proliferative subtype is a well-defined subtype of gastric cancer characterized by *p53* mutations, DNA hypermethylation, as well as activated E2F, Myc and Ras oncogenic pathways [73]. H19 is concordantly stimulated by tumor suppressor P53 [74] and onco-protein c-Myc [75].

**Figure 4:**
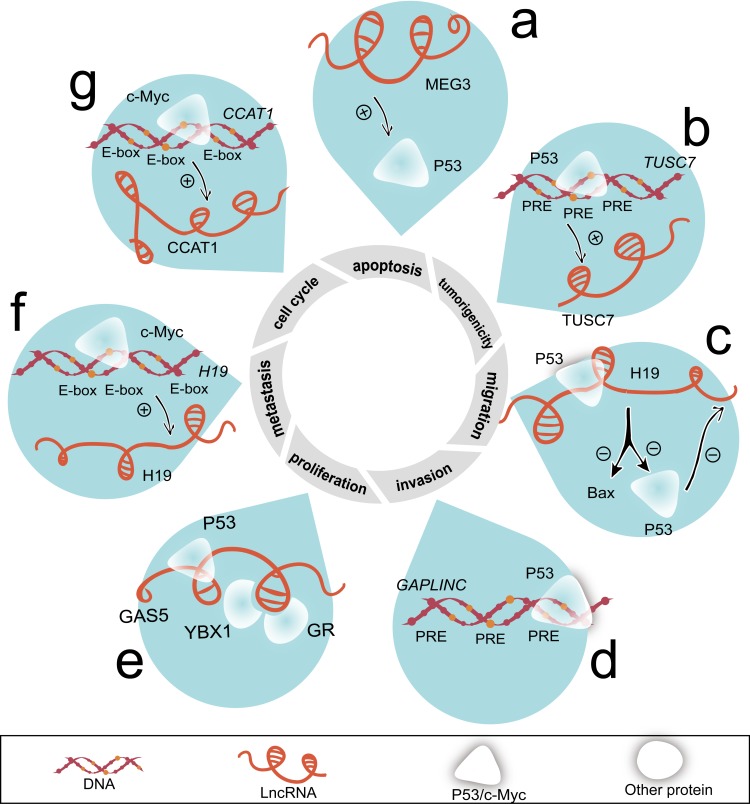
LncRNAs regulate cell proliferation, cell cycle, apoptosis, invasion, migration, metastasis, and tumorigenicity mediated by P53 and c-Myc **a.** MEG3 accelerates P53 protein level. **b.** TUSC7 is transcriptionally activated by P53, which binds with PREs of its promoter. **c.** Combining with P53, H19 partially decreases P53 activity and BAX expression. **d.** P53 occupies the PREs on the promoter of GAPLINC. **e.** GAS5 combines with P53, YBX1 and GR. **f.** Using E-box, H19 is treated as a target of c-Myc. **g.** The abundance of CCAT1 is accelerated by c-Myc. c-Myc occupied the E-box on CCAT1 promoter region. Abbreviations: BAX, BCL2-associated X protein.

Recent study identified several lincRNA loci enriching consensus P53 responsive elements (PREs) [76]. Meanwhile, ten differentially expressed lncRNAs potentially manipulate the P53 signaling pathway in gastric cancer [77]. Several lncRNAs including MEG3 (Figure 4a), TUSC7 (Figure 4b), and H19 (Figure 4c) execute wild type P53 instructions and also serve as regulators of wild type P53 [47, 74, 78, 79]. Moreover, the folding of full length MEG3 is crucial for inducing significant increase of P53 levels [80]. GAPLINC promoter contains mutant P53-binding motif (Figure 4d). Growth arrest-specific 5 (GAS5) bounds to P53/ Y box binding protein 1 (YBX1) complex and upregulates P21 expression [81] (Figure 4e).

Myc protein contains a basic DNA binding domain that binds to E-box DNA recognition fragment (CACGTG) [82]. CpG islands and pre-acetylated state of chromatin enable high affinity of sites bound to c-Myc [83]. c-Myc is overexpressed in 43% gastric cancer patients [84]. Microarray results indicated that 1244 lncRNAs are directly activated by c-Myc [85]. Remarkably, two lncRNAs, H19 (Figure 4f) and colon cancer associated transcript 1 (CCAT1) (Figure 4g) are transcriptionally activated by c-Myc in gastric cancer [75, 86]. In addition, the active regulatory region of the CCAT1 site occurs physically interaction with *c-Myc* enhancer region [87].

### Interaction with other proteins

With the exception of the direct interaction with proteins, how do lncRNAs regulate the levels of proteins is arresting. GAS5 positively influences YBX1 protein stability without increasing its transcription [81]. The putative stem-loop structure formed by exon 12 of GAS5 is responsible for its interplay with YBX1 (Figure 2d). YBX1 possesses the capacity of complexing with IGF2BP1 [88], which combines with GHET1 and prevents mRNA degradation [33]. The exon 12 is a GAS5′s predominant structural domain for mimicking binding domain of glucocorticoid receptor (GR) [89]. GAS5, GR, YBX1, and P53 may collaborate as complex to achieve cell cycle regulation [90] (Figure 2d).

H19 is found bound with Isthmin 1 (ISM1) [45] and positively regulates its expression (Figure 2e). ISM1 is a factor promoting endothelial cell survival and cell death synchronously [91]. This may explain why H19's role converts between multiple cancers. H19's high expression level enforces the oncogenic progress of gastric cancer cells; while in hepatocellular carcinoma (HCC), it is recognized as tumor suppressor [92]. Additionally, H19-derived miR-675 may succeed in causing the alterations of ISM1 [45].

There are also inhibitory effects of lncRNAs on the binding proteins, such as nc886 on double stranded RNA-dependent protein kinase (PKR) (Figure 2f) and HOTAIR on poly(C) binding protein 1 (PCBP1) (Figure 2g). PKR recognizes single strand nucleotide sequence in the central region of nc886 [93], which deviates from canonical PKR ligands with abundant hairpin structures [94]. In this way, nc886 displays its anti-apoptotic ability in a cell type dependent manner [95] (Figure 2f). PCBP1 inactivates the AKT pathway to against metastatic progression [96]. This finding *per se* matches the potential of HOTAIR in metastasis in gastric cancer.

## CONCLUSIONS

LncRNAs are characterized by the complexity of their mechanisms. We summarized lncRNAs’ interaction with DNA, RNA, and proteins in gastric cancer occurrences.

It is tempting to speculate that a multitude of lncRNAs may interrupt definitive steps in numerous tumor suppressive and oncogenic pathways. The uncovering of the underlying mechanisms of lncRNAs may benefit our understanding of gastric cancer's pathogenesis.
